# The next step in health behavior research: the need for ecological moderation analyses - an application to diet and physical activity at childcare

**DOI:** 10.1186/1479-5868-11-52

**Published:** 2014-04-17

**Authors:** Jessica S Gubbels, Dave HH Van Kann, Nanne K de Vries, Carel Thijs, Stef PJ Kremers

**Affiliations:** 1 Department of Health Promotion, Maastricht University, PO Box 616, Maastricht, MD, 6200, the Netherlands; 2 NUTRIM, School for Nutrition, Toxicology and Metabolism, Maastricht University, PO Box 616, Maastricht, MD, 6200, the Netherlands; 3 Academic Collaborative Centre for Public Health Limburg, Regional Public Health Service, PO Box 2022, Geleen, HA, 6160, the Netherlands; 4 Caphri, School of Public Health and Primary Care, Maastricht University, PO Box 616, Maastricht, MD, 6200, the Netherlands; 5 Department of Epidemiology, Maastricht University, PO Box 616, Maastricht, MD, 6200, the Netherlands

**Keywords:** Childcare, Diet, Ecological perspective, Environment, Interaction, Meso-system, Moderation, Overweight, Physical activity, Preschoolers

## Abstract

**Background:**

The ecological perspective holds that human behavior depends on the interaction of different environmental factors and personal characteristics, but it lacks validation and operationalization. In the current paper, an ecological view was adopted to examine the interactive impact of several ecological systems on children’s dietary intake and physical activity at childcare or similar facilities. The ecological view was operationalized into three types of interaction: 1) interaction between types of childcare environment (physical, social, political, economic); 2) interaction between micro-systems (the childcare and home environment) in meso-systems; and 3) interaction between childcare environment and child characteristics. The predictive value of each of these interactions was tested based on a systematic review of the literature.

**Discussion:**

Several studies support the hypothesis that the influence of the childcare environment on children’s physical activity and diet is moderated by child characteristics (age, gender), but interaction between environmental types as well as between micro-systems is hardly examined in the field of behavioral nutrition and physical activity. Qualitative studies and general child development research provide some valuable insights, but we advocate quantitative research adopting an ecological perspective on environmental influences.

**Summary:**

Empirical studies operationalizing a true ecological view on diet and physical activity are scarce. Theorizing and assessment of interaction is advocated to become common practice rather than an exception in behavioral nutrition and physical activity research, in order to move the field forward.

## Background

It is striking that studies into proximal determinants of many human behaviors have almost exclusively been limited to examination of separate elements (environmental factors, personal factors), each assumed to influence behavior separately. Studies often focus on specific aspects of a problem, losing track of the ‘bigger picture’. To illustrate, in a previous debate paper in the *International Journal of Behavioral Nutrition and Physical Activity*, Brug and colleagues [1] advocated, among others, the ‘Intervention Mapping’ approach. This approach forces its users to specify each of the determinants of a certain behavior, and think of separate change strategies addressing each of those specific factors [2]. Although we acknowledge and underline the importance of a systematic approach to understanding and changing health behavior, such ‘separate elements’ approaches and theories fail to acknowledge that the complete picture is ‘more than the sum of its parts’. Admittedly, Intervention Mapping does not explicitly exclude interaction between specific determinants, but the approach does emphasize linear bivariate relationships. In line with this, Brug et al. [1] stated that ‘what we really need are not studies that highlight the importance of individual factors, social factors *or* physical environmental factors in shaping nutrition and PA behaviors. We need more studies that integrate potential determinants at the environmental level *and* the individual levels’. Resnicow and Vaughan [3] responded with a paper in which they questioned the current cognitive-rational paradigm in health promotion, and instead proposed a chaotic view. One of the propositions of this chaotic view is that the elements of a complex system interact in a non-linear fashion in explaining behavior [3]. The implications of this view for health promotion practice are, however, considered limited [4], perhaps because the view is difficult to operationalize in determinants studies and interventions.

In a further contribution in the debate on the usefulness of theories in the field of behavioral nutrition and physical activity, Kremers and colleagues [5] integrated the different views on health behavior theory, creating the Environmental Research framework for weight Gain prevention (EnRG framework). This framework combines both personal determinants *and* environmental determinants, specifying the interactive nature of their relationship. As such, the framework may be a useful addition to traditional models that focus on either environmental *or* personal determinants of behavior.

Another, much discussed theoretical framework which integrates personal and environmental determinants of behavior is the ecological framework [6,7]. Although a growing number of studies now recognize the multivariate and multilevel structure of determinants of behavior, their analysis often stops right there. Various studies examine impressive, evidence-based, integrated multilevel lists of contributors to childhood obesity, claiming to apply an ecological view (e.g. [8-10]). However, integration is not synonymous to interaction. The relationships *between* these contributors are often ignored in these studies. By doing so, they disregard the assumption of interaction between behavioral determinants that is right at the core of a true ecological perspective [11]. In an attempt to further contribute to the debate on the usefulness of theories in the field of energy balance-related behaviors (EBRBs), the current paper aims to operationalize and validate a *true* ecological perspective.

### An ecological perspective

Determinants of behavior cannot be viewed in isolation. They influence not only the behavior at interest but also each other, and it is their combined influence that determines human behavior, forming a complex system [12]. This in line with an ecological ‘systems’ view of environmental influences on human behavior [6,7]. Ecological models propose an interaction between the environment and the individual, as well as interaction between elements within the environment [11]. Conceptually, this means that individuals with different characteristics or in different contexts react differently to similar influences [13]. In other words, environmental influences on EBRBs cannot be generalized, but are person and situation specific.

The ecological perspective needs validation and operationalization [11], but hypotheses remain vague and actual adoption in practice is lacking [14]. Even theoretical applications to dietary behavior and/or physical activity such as the Ecological Model of Physical Activity (EMPA) lack specificity and operationalization. As the originators of the EMPA acknowledge, specific characteristics of relationships among the factors in their model need to be identified [11]. The ecological perspective thus remains an idea in need of true operationalization and validation. As a first step towards this goal, we reviewed the current literature to examine the state-of-the-art in adopting an ecological view to study the interactive impact of several ecological systems on children’s dietary intake and physical activity.

### A practical example: EBRBs at childcare

Validation of the ecological perspective using young children as a sample case is a feasible starting point. Firstly, although incorporating the environment in explaining behavior is important for all human behaviors, it becomes even more important regarding children. Children’s behavior is largely unreasoned, unplanned, and environment-driven, making traditional cognitive behavior explanation models mostly unsuitable for this age group [15]. Secondly, the variety and complexity of environments increases throughout life: whereas adults encounter an enormous variety of environments (e.g. work, home, sports clubs), young children encounter only a handful at the most, the main ones being home and childcare [16]. The application to children’s behavior should thus be seen as a starting point, allowing further extrapolations and generalizations to other settings, ages and behaviors. Furthermore, EBRB habits are often formed at a young age and maintained during later life [17,18], making it essential to target EBRBs in early childhood.

Various studies have shown an increased overweight risk in children attending childcare (e.g. [19-22]). Childcare and similar facilities (e.g. pre-school, kindergarten, day-care) could play an important role in childhood overweight prevention [23]. A review by Larson and colleagues provides a detailed overview of existing studies on these environmental determinants of EBRBs at childcare [24]. This review showed that the majority of the existing studies reported on either child behavior *or* the childcare environment, failing to examine the association between both. An example of an environmental determinant of EBRBs at childcare is childcare staff behavior, which has been found to be important for shaping children’s dietary intake at childcare [25,26], as well as their physical activity level [27]. Increased PA has further been linked to for instance the availability of play equipment and PA opportunities [27-30], a ‘natural’ environment [31], smaller group size [27,32,33], child-initiated instead of staff-initiated play [32] and prompts by peers [27]. As we will see in the next paragraph, other important determinants of EBRBs such as the family context and characteristics of the child itself, and the interplay between all these factors are a necessary addition.

### Application of the ecological framework to childcare

**Figure 1:**
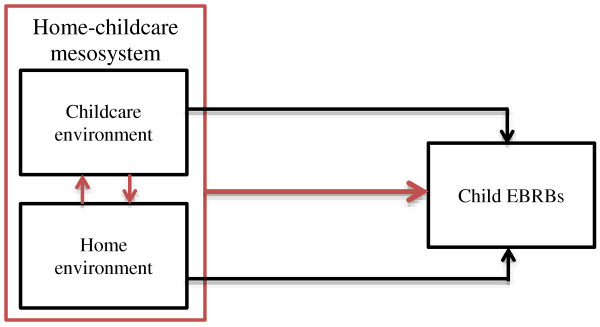
Interaction between home environment and childcare environment, forming a meso-sytem influencing child EBRBs (red arrows).

Studies into determinants of children’s EBRBs have, in line with studies into many other human behaviors, almost exclusively been limited to examination of separate ‘micro-environments’ (i.e., the immediate settings within which individuals interact), e.g. focusing on the influence of either childcare *or* home influences. However, in practice home and childcare environments interact with each other in influencing children’s EBRBs and weight status [12,34] (see Figure 1). The influence of the childcare environment thus depends on what happens at home, and vice versa, in line with the ecological view [6,7]. Bronfenbrenner, the founder of the ecological perspective, has defined interaction between micro-systems as the ‘meso-system’ [35].

**Figure 2:**
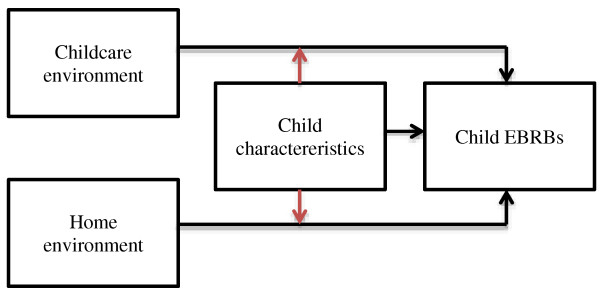
Moderation of the relationship between environment and child EBRBs by child characteristics (red arrows).

Another moderating factor that has mostly been overlooked is the influence of the child itself (see Figure 2). Ecological models include a notion of the importance of a good ‘fit’ between individuals and their environment [36]. Children should be seen as active agents, shaping and interpreting their environment [37]. Their individual characteristics might influence how well they can adapt to the environment. In line with this, the ecological system stresses the importance of synergy between individuals and their environment [11]. Moreover, this concurs with guidelines for the study of correlates of EBRBs, which state that such research should attempt to integrate personal and environmental correlates of these behaviors [38].

**Figure 3:**
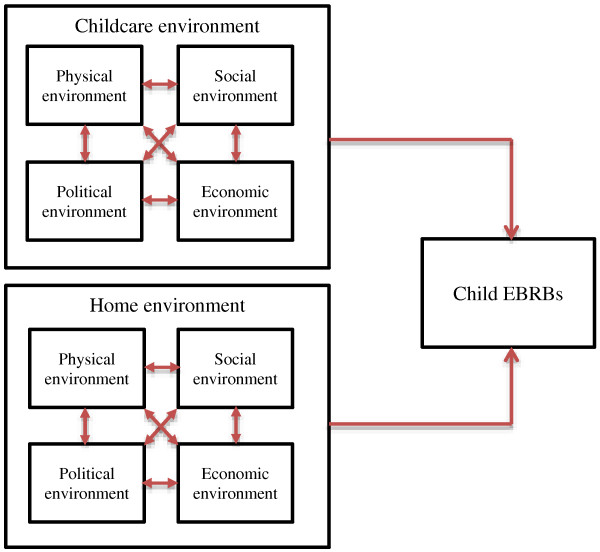
Interaction between types of environments in influencing child EBRBs (red arrows).

Furthermore, ecological models propose that within each environmental setting there are multiple types of environment [6]. The ANGELO framework [39] operationalizes the environment through distinguishing between four types: the physical environment (what is available, e.g. play equipment), the social environment (what are the attitudes/beliefs of important others, e.g. the general parenting/supervision climate), the political environment (what are the rules, e.g. about child’s EBRBs), and the economic environment (what are the relative costs, e.g. relative budget of the childcare center). The ecological view presumes that these environmental types interact in determining child behavior [6] (see Figure 3).

From the ecological viewpoint, effects of existing overweight prevention interventions focusing on single determinants of EBRBs at childcare are probably reduced by the moderating influences of other factors, not taken into account in these interventions. The ecological systems view [6,7] furthermore needs additional validation in multiple settings [5,6,11,40]. In the current paper, we will discuss the validity of the ecological framework, by applying it to the childcare setting. We have operationalized the ecological framework into three types of interactions: 1) the interaction between types of environment (physical, social, political, economic) at childcare; 2) the interaction between micro-systems (the childcare and home environment) in meso-systems; and 3) the interaction between environmental factors at childcare and child characteristics. These hypotheses will be tested based on a systematic review of the literature.

A systematic review of the literature performed by two independent researchers. Databases PubMed and PsycInfo were searched using a combination of keywords referring to childcare, environment and nutrition or physical activity. In addition, snowball sampling was used. For the current article, we define childcare as organised care for children where parents leave their child for more than a few hours a day with (semi-)professional staff. This includes child daycare and pre-school, but excludes homecare by a relative, babysitter or nanny, and care in settings that entirely replace daily home like boarding school and foster home.

All English papers published between January 1990 and May 2013 were included. After removing duplicates, this resulted in a total of 4069 papers. After title screening, 292 papers remained for abstract screening, from which 44 papers were selected for full text screening. A total of 25 quantitative studies that examined an association between the childcare environment and either children’s dietary intake [25,26,41-46] or physical activity [27-33,47-56] were identified. However, only seven of these studies included some examination of interaction between types of environment, between the childcare environment and the home environment or between environmental factors at childcare and child characteristics [27,29,31,33,46,52,56] (Table 1). Six studies regarded physical activity; only one study examined dietary intake. The environmental features assessed were all physical or social.

**Table 1:** Overview of the general characteristics examining the ecological view in the childcare settings, and findings regarding the interaction between childcare environmental factors and child characteristics

**Study**	**Sample**	**Study type**	**Predictors**		**Outcome variable**	**Associations**		
			***Environmental factors***	***Child charac-teristics***		***Direct environmen-tal effects***	***Indication interaction effects***	***Interaction effects***
Boldemann et al., 2006 [29]	* N = 197	Obs.	1. Physical environment:	* Gender	* Average step count.	1a: +	Subgroup analyses	* Gender:
	* 4–6 y		a. Environment category (outdoors)		* Assessed by pedometers	1b: 0		1a: F: 0, M: +
	* 11 preschools		b. Indoor space					
	* Sweden		2. Political environment:		* Indoors and outdoors together	2a: +		
			a. Outdoor education					
Cardon et al., 2008 [31]	* N = 783	Obs.	1. Physical environment:	* Gender	* Average step count	Not assessed (only subgroup analyses reported)	Subgroup analyses	* Gender:
								1c: F: 0, M: −
	* 4–5 y,		a. Aiming equipment		* Assessed by pedometer			2a: F: −-, M: −
								2b: F: −, M: 0
	av. = 5.3 y		b. Playing equipment					(3a: F: −, M: − )
	* 39 preschools		c. Soft ground surface		* Outdoors			
	* Belgium		d. Markings					
			e. Vegetation					
			f. Height differences					
			g. Toys					
			2. Social environment:					
			a. Children/m^2^					
			b. Number of supervisors					
			3. Political environment:					
			a. Recess duration					
Gubbels et al., 2011 [25]	* N = 175	Obs.	1. Physical environment:	* Gender	* Average PA intensity level	1a: + (I/O)	Moderation analyses and post-hoc analyses	* Gender:
						2a: 0		2a (I): F: −, M: 0
			a. Sum score activity opportunities (EPAO)	* Age	* Assessed by observation (OSRAC-P)	2b: + (O)		2a (O): F: 0, M: +
	* 2–3 y, av. = 2.6 y		2. Social environment:			2c: 0		2b (I): F: −, M: 0
	* 9 childcare centers		a. PA discouraging prompts peers			2d: + (I/O)		2b(O):F: +, M: ++
	* Netherlands		b. PA promoting prompts peers		* Indoors (I) and outdoors (O) separately	2e: − (I/O)		* Age:
						2f: − (I)		2e (O): 2y: 0, 3y: −
			c. PA discouraging prompts supervisors					2f (I): 2y: −-, 3y: −
			d. PA promoting prompts supervisors					
			e. Group size peers					
			f. Group size supervisors					
Hannon & Brown, 2008 [27]	* N = 64	Int.	Physical environment	* Gender	* Time spent at PA intensity levels (sedentary, light, moderate, vigorous)	Intervention effect: +	Moderation analyses and post-hoc analyses	* Age:
								Moderate Activity: 3y:+++, 4y:++, 5y:+
	* 3–5 y		Activity-friendly playground intervention, providing children with various physical activity facilities/equipments (e.g. hurdles, hoops, tunnels, balance beams, balls)	* Age				
	* 1 preschool				* Assessed by accelerometer			Vigorous Activity: 3y:+, 4y:+, 5y:++
					* Outdoors			
	* USA							
McKenzie et al., 1997 [49]	* N = 287	Obs.	None (outcome variable comprises both environmental characteristic (PA prompt) and physical activity)	* Gender	* Compliance (increasing/maintaining MVPA) to PA prompts.	+ (prevalence compliance to PA prompts is 89.5%)	Subgroup analyses	* Gender:
	* av. age = 4.4 y							
	* 63 preschools							
	* USA							F: ++, M: +
				* Ethnicity (European-American vs. Mexican-American)				
					* Assessed by observation.			
					* Outdoors			
Van Cauwenberghe et al., 2012 [53]	* N = 128	Int.	Physical environment	* Gender	* Time spent at PA intensity levels (sedentary, LMVPA, MVPA.).	+	Subgroup analyses	* Gender:
	* 4–6 y,		Play space per child					F: ++, M: +
	av. = 5.1 y							
	* 4 preschools							
	* Belgium							
					* Assessed by accelerometer			
					* Outdoors			
Zuercher & Kranz, 2012 [43]	* N = 54	Int.	Physical environment	* Age	* Dietary fiber intake	+	Subgroup analyses	* Age:
								2-3y: ++, 4-5y: +
					* Assessed by plate weighing			
			Availability of high fiber lunch items					
	* 2-5 y							
	* 1 childcare center							
	* USA							

+ = positive association, − = negative association, 0 = no association. ++ vs. + and -- vs. - = stronger pos/neg. association.

*Abbreviations: av., Average; EPAO, Environment and Policy Assessment Observation*[80]*; F, Female; I, Indoor; int., Intervention study; LMVPA, Light, Moderate and Vigorous Physical Activity; M, Male; MVPA, Moderate to Vigorous Physical Activity; neg., Negative; O, Outdoor; obs., Observational study; OSRAC-P, Observation System for Recording Physical Activity in Children – Preschool version*[81]*; PA, Physical activity; pos., Positive; y, Years old.*

## Discussion

### Interaction between types of environment

The first type of interaction, between types of environment (Figure 3), was examined by only one study [27]. This study examined the influence of social and physical environmental factors on physical activity at childcare, and whether there was an interaction between both (Table 1). Moderation analyses showed that the positive influence of activity opportunities (physical environment) on outdoor physical activity was only present when children were engaged in an activity together with multiple peers (social environment). Children playing alone or with one other peer seemed unaffected by the presence of activity opportunities. This study thus showed some evidence for interaction between the physical and social childcare environment, supporting the ecological framework. This is however the only study we found that provides such indications. Studies examining such interactions in influencing dietary intake were not found, nor did we find any studies examining interaction between or with any of the other types of environment (e.g. interaction between the political and economic childcare environment). Further research would thus be needed to examine this element of the ecological view.

### Interaction between microsystems

With regard to interaction between the childcare environment and the home environment in influencing children EBRBs, (Figure 1) no quantitative studies examining this were found. Qualitative research nevertheless provides strong indications that such interaction exists. Several qualitative studies among childcare workers highlighted the importance of support of and communication with parents for promoting sufficient physical activity and healthy dietary intake at childcare [57-60]. Furthermore, parents were explicitly mentioned by childcare staff as an obstacle to physical activity, as they tried to restrict children’s activities at the childcare centers (e.g. limiting time spent outdoors, letting children wear clothes unsuitable for physical activity [59-61]), or expressed other priorities for their child at childcare, such as academic performance and safety concerns [62]. In addition to parents influencing physical activity in the childcare setting, childcare workers also indicated interaction the other way around: they advised parents regarding children’s physical activity and nutrition and were aware that they could (or perhaps should) be a role model for parents [60,63]. In line with this, the Institute of Medicine has suggested that it is the role of ‘non-home environments’ such as childcare to support parents in their efforts to promote physical activity and healthy eating in their children [64]. Parents also indicated that childcare practices influenced the home situation [65], and some parents also actively sought advice from the childcare center on EBRB-related parenting [58]. Discontinuity between the childcare setting and home was furthermore perceived as hampering sufficient physical activity at childcare [57].

General child development research provides some further indications concerning the interaction between childcare and home in influencing children’s behavior. Parents and childcare staff often have different child-rearing attitudes, values and practices [66,67], and such inconsistency affects children’s wellbeing negatively [66,68]. This is implied by the ecological framework: the stronger the link between two micro-systems, the better the child’s outcomes [69]. The problem is that although the child participates fully in these two micro-systems with different demands (forming a meso-system), parents and childcare staff do not have such a direct link with each other’s micro-system [69]. This link could be created through communication: Communication between home and childcare creates synergism and consistency, supporting optimal child development [70]. Parents and supervisors should for instance make agreements on the foods that should be offered to the child.

Some research, however, has shown that discontinuity between micro-environments is not necessarily bad, especially when a high-quality micro-environment (e.g. a high-quality childcare center) moderates (i.e. diminishes) the adverse effects of a low-quality micro-environment (e.g. a disadvantaged home; [70]). Some further argue that children might benefit from different experiences, and that optimal adaptation to the environment is more important than structural similarity [70], which is in line with our final hypothesis, regarding interaction between the environment and child characteristics.

We feel that the findings of qualitative studies and studies regarding general child development provide sufficient rationale to examine the home/childcare meso-system hypothesis in quantitative studies for EBRBs. Such studies would be quite extensive and need to be thoroughly designed, which might very well be the reason that this hypothesis has not yet been studied in quantitative studies to date. Applying the ecological framework has large consequences for the research designs to be used, including a simultaneous assessment of both the behaviors and the environments, in all relevant settings (home and childcare in the current example), using the same instruments in all settings, in the same persons (children in the current example). Furthermore, sample size will have to be increased, in order to be able to test for interaction.

### Interaction between environmental influences and child characteristics

In contrast to the first two types of interactions within the ecological framework, there is relatively convincing evidence for a moderating role of child characteristics in the relationship between the childcare environment and children’s EBRBs [27,29,31,33,46,52,56] (see Table 1, Figure 2). The child characteristics that have been examined as potential moderators of environmental influences are gender, age and ethnicity. Indications for existence of such moderation were found for age and gender, but not for ethnicity.

Four out of the five studies that examined gender as a moderator of environmental influences, did actually find indications of the existence of such interaction. In general, boys seemed to be more strongly affected by the physical environment than girls: boys’ activity levels were positively associated with the quality of the outdoor environment [31], and negatively with soft ground surfaces [33], while in girls both associations were absent [31,33]. However, girls profited more from an intervention increasing the available play space per child [55]. Social environmental influences were moderated by gender as well. Girls were more negatively influenced than boys by increasing group size [33]. Girls and boys further responded differently to prompts. McKenzie et al. [52] reported higher compliance to activity promoting prompts from supervisors in girls, while Gubbels and colleagues [27] found that the association between activity promoting prompts from peers and activity levels was stronger in boys. With regard to activity discouraging prompts, girls responded more negatively than boys [27].

Children’s age also seems to interact with environmental influences in influencing both physical activity and dietary intake. With regard to the physical environment, older children were found to mainly increase their *vigorous* activity in response to an activity-friendly playground intervention, while younger children mainly increased their *moderate* activity [29]. With regard to social environmental factors, older children’s activity levels were more negatively affected by the number of peers present, while younger children were more negatively affected by the number of supervisors [27]. As regards dietary intake, availability of high fiber lunch items (physical environment) showed a stronger positive effect in younger children compared to older children [46]. In line with these moderation effects reported in quantitative studies, childcare supervisors have also indicated in qualitative research that children have different needs with regard to physical activity at different ages, and that this age diversity is considered a constraint on realizing sufficient physical activity for all children [60,61].

The fact that child gender and age influence the effect the childcare environment has on EBRBs, indicates that different children need different environmental interventions to stimulate healthy EBRBs. Childcare supervisors could for instance be made aware of these different responses of children, and should be educated about how physical activity can be promoted in all children [33]. This could potentially increase the effectiveness of such interventions, as well as help to address the disparity observed in health-related behaviors between children [71]. In addition, these moderating processes have implications for research in the area of EBRBs. Ignoring moderation might lead to oversimplified conclusions. For example, Dowda and colleagues [49] found that the number of children present was not significantly associated with children physical activity levels. However, the authors of this study did not examine possible moderation of this association by child characteristics. Two of the studies included in the current review showed that association between number of children present and physical activity is moderated by gender [33] and age [27]. The reported association by Dowda et al. [49] could thus have been nullified in the analyses because of the lack of an examination of moderation by child characteristics.

It would be interesting to examine other child characteristics in addition to gender and age as moderators of environmental influences as well. A review of moderators of environmental intervention effects on diet and activity in youth (aged 3–18 years), indicated that in addition to gender and age, also ethnicity moderates environmental effects on behavior [72]. The moderating influence of ethnicity was examined in one of the included studies at childcare, but this was not confirmed [52]. Other child-related moderators found in settings other than the childcare center include children’s temperament or personality tendency and weight status [73-75]. These additional potential moderators should also be examined at childcare.

### Quality of the studies

The quality of the studies reporting indications of interactions between the childcare environment and child characteristics differs greatly. Two studies statistically tested the interaction between the environment and several child characteristics selected based on previous research [27,29]. Statistical testing of the significance of interaction terms is considered the most appropriate method for examining interaction [76]. The other studies performed subgroup analyses without testing the significance of interaction terms, basing their subgroup analyses solely on theoretical reasons, for instance [31,33,49,52,56]. Also methodological quality differed, with large variation in for instance sample sizes and quality of assessment of both predictors and outcomes (see Table 1). The quality of the study by McKenzie et al. [52] for making inferences with regard to the current hypotheses was further debatable, as the authors did not assess the environment and activity separately, but included a variable that incorporated both: compliance to physical activity prompts. The inconsistent quality of the existing studies provides an additional stimulus for further high quality research regarding the validity of the ecological framework.

## Summary and conclusion

The current paper examined the validity of the ecological view of environmental influences on human behavior, for EBRBs in the childcare setting specifically [6,7]. In general, the evidence for the validity of the ecological framework for EBRBs at childcare is scarce, especially regarding dietary behavior. Most evidence was found for moderation of environmental influences by child characteristics (i.e. child age and gender). The small number of studies applying the ecological view limits our insight into the complex interplay of environmental influences on child EBRBs. As such, this paper should be considered as a call for studies that apply the ecological approach in the examination of environmental influences on energy balance-related behaviors in the childcare setting.

Given that the studies that did examine the ecological framework appear to confirm its validity, the lack of support for this framework could probably be attributed to the lack of research examining the interactions. Reasons for a lack of such examinations might be found in the complexity inherent to such studies, with high demands regarding for instance the study’s sample size, design and measurement instruments. These requirements present us with quite a challenge. We encourage researchers in the area of EBRBs to cooperate with statisticians and other research areas in order to explore innovative ways of dealing with the methodological and statistical issues we are confronted with, in order to move the EBRB field forward in terms of the methodology and statistics used as well. If we don’t, our methodology will eventually lag behind on our theory.

The lack of studies applying the ecological framework might perhaps also be attributed to publication bias, as some researchers might have failed to report non-significant findings. Nonetheless, also qualitative research and research in the area of general child development, provide clear indications for the validity of the ecological framework, and thus justify further quantitative research on this topic. Furthermore, quantitative studies examining the ecological framework are also lacking when looking at other age groups: although relatively many studies examine moderation of environmental influences by personal factors (e.g. [77,78]), few studies examine interaction within and between environments (e.g. [79]). This is in line with the call of various authors to further validate the ecological framework in various settings [5,6,11,40]. Moreover, effects of existing overweight prevention interventions focusing on single determinants of EBRBs at childcare may be limited by the moderating influences of other factors not taken into account, which further stresses the urgency of such research.

In the case of childcare specifically, Bradley stated that in view of the wide use and presumed importance of childcare, it is striking how little research has been devoted to the family/childcare interaction; part of this neglect probably derives from the complexity and dynamics of the system [34]. Proper examination of the whole system from a true ecological perspective requires extensive, well designed, longitudinal studies. However, we cannot continue to neglect these interactions, ignoring the fact that what we find is not the complete picture, or simply wrong. The effectiveness of existing interventions focusing on single determinants of health behavior is probably suboptimal by not taking moderation into account, wasting valuable time and money in battling lifestyle-related health issues such as obesity. Given the gravity of these diseases, we are obliged to take the next step, and apply a *true* ecological perspective to health behavior. Assessment of moderation should thus become common practice rather than an exception in behavioral nutrition and physical activity research [72], including research in the childcare setting. We all know that we should: an umbrella review of 36 reviews summarizing the influence of the physical environment on physical activity, revealed that the most cited suggestion for future research was to examine moderators of environmental influences [40]. Let’s take the next step.

## Abbreviations

ANGELO: Analysis Grid for Environments Linked to Obesity; EBRBs: Energy balance-related behaviors.

## Competing interests

The authors declare that they have no competing interests.

## Authors’ contributions

All authors made substantial contributions to the design of the study. JSG and DHHVK performed the literature review, and JSG wrote draft versions of the manuscript. All authors were involved in critically revising the manuscript, and have given their approval for the submitted manuscript. All authors read and approved the final manuscript.

## References

[B1] Brug J Oenema A Ferreira I Theory, evidence and Intervention Mapping to improve behavior nutrition and physical activity interventions Int J Behav Nutr Phys Act 2005 2 2 10.1186/1479-5868-2-2 15807898 PMC1087867

[B2] Bartholomew LK Parcel GS Kok G Gottlieb Intervention mapping: designing theory- and evidence-based health promotion programs 2001 New York: McGraw-Hill

[B3] Resnicow K Vaughan R A chaotic view of behavior change: a quantum leap for health promotion Int J Behav Nutr Phys Act 2006 3 25 10.1186/1479-5868-3-25 16968551 PMC1586207

[B4] Brug J Order is needed to promote linear or quantum changes in nutrition and physical activity behaviors: a reaction to ‘A chaotic view of behavior change’ by Resnicow and Vaughan Int J Behav Nutr Phys Act 2006 3 29 10.1186/1479-5868-3-29 16982008 PMC1584250

[B5] Kremers SP de Bruijn GJ Visscher TJ van Mechelen W de Vries NK Brug J Environmental influences on energy balance-related behaviors: a dual-process view Int J Behav Nutr Phys Act 2006 3 9 10.1186/1479-5868-3-9 16700907 PMC1481572

[B6] Friedman SL Wachs TD Measuring Environment Across the Life Span. Emerging Methods and Concepts 1999 Washington: American Psychological Association

[B7] Wachs TD The Nature of Nurture 1992 Newbury Park, CA: Sage

[B8] Boonpleng W Park CG Gallo AM Corte C McCreary L Bergren MD Ecological influences of early childhood obesity: a multilevel analysis West J Nurs Res 2013 35 742 759 10.1177/0193945913480275 23493675

[B9] Davison KK Jurkowski JM Lawson HA Reframing family-centred obesity prevention using the Family Ecological Model Public Health Nutr 2013 16 1861 1869 10.1017/S1368980012004533 23089267 PMC5500251

[B10] Hawkins SS Cole TJ Law C An ecological systems approach to examining risk factors for early childhood overweight: findings from the UK Millennium Cohort Study J Epidemiol Community Health 2009 63 147 155 18801795 10.1136/jech.2008.077917 PMC2678539

[B11] Spence JC Lee RE Toward a comprehensive model of physical activity Psychology of Sport and Exercise 2003 4 7 24 10.1016/S1469-0292(02)00014-6

[B12] Kremers SP Theory and practice in the study of influences on energy balance-related behaviors Patient Educ Couns 2010 79 291 298 10.1016/j.pec.2010.03.002 20371159

[B13] Wachs TD Necessary but not Sufficient: The Respective Roles of Single and Multiple Influences on Human Development 2000 Washington: American Psychological Association

[B14] Huang TT Drewnoski A Kumanyika S Glass TA A systems-oriented multilevel framework for addressing obesity in the 21st century Prev Chronic Dis 2009 6 A82 19527584 PMC2722412

[B15] Bernstein DA Penner LA Clarke-Stewart A Roy EJ Psychology 2003 Boston: Houghton Mifflin Company

[B16] Bos W Huynen B Gebruik en kosten van kinderopvang, 2006–2008 Sociaaleconomische Trends, 3e Kwartaal 2010 2010 Den Haag/Heerlen: Centraal Bureau voor de Statistiek

[B17] Reilly JJ Jackson DM Montgomery C Kelly LA Slater C Grant S Paton JY Total energy expenditure and physical activity in young Scottish children: mixed longitudinal study Lancet 2004 363 211 212 10.1016/S0140-6736(03)15331-7 14738795

[B18] Kelder SH Perry CL Klepp KI Lytle LL Longitudinal tracking of adolescent smoking, physical activity, and food choice behaviors Am J Public Health 1994 84 1121 1126 10.2105/AJPH.84.7.1121 8017536 PMC1614729

[B19] Gubbels JS Kremers SPJ Stafleu A Dagnelie PC de Vries NK van Buuren S Thijs C Child-care use and the association with body mass index and overweight in children from 7 months to 2 years of age Int J Obes (Lond) 2010 34 1480 1486 10.1038/ijo.2010.100 20498654

[B20] Benjamin SE Rifas-Shiman SL Taveras EM Haines J Finkelstein J Kleinman K Gillman MW Early child care and adiposity at ages 1 and 3 years Pediatrics 2009 124 555 562 10.1542/peds.2008-2857 19651579 PMC3049895

[B21] Maher EJ Li G Carter L Johnson DB Preschool child care participation and obesity at the start of kindergarten Pediatrics 2008 122 322 330 10.1542/peds.2007-2233 18676550

[B22] Geoffroy MC Power C Touchette E Dubois L Boivin M Seguin JR Tremblay RE Cote SM Childcare and overweight or obesity over 10 years of follow-up J Pediatr 2013 162 753 758 e1 10.1016/j.jpeds.2012.09.026 23140878

[B23] Story M Kaphingst KM French S The role of child care settings in obesity prevention Future Child 2006 16 143 168 10.1353/foc.2006.0010 16532662

[B24] Larson N Ward DS Neelon SB Story M What role can child-care settings play in obesity prevention? A review of the evidence and call for research efforts J Am Diet Assoc 2011 111 1343 1362 10.1016/j.jada.2011.06.007 21872698

[B25] Gubbels JS Kremers SP Stafleu A Dagnelie PC de Vries NK Thijs C Child-care environment and dietary intake of 2- and 3-year-old children J Hum Nutr Diet 2010 23 97 101 10.1111/j.1365-277X.2009.01022.x 19943841

[B26] Hughes SO Patrick H Power TG Fisher JO Anderson CB Nicklas TA The impact of child care providers’ feeding on children’s food consumption J Dev Behav Pediatr 2007 28 100 107 10.1097/01.DBP.0000267561.34199.a9 17435460

[B27] Gubbels JS Kremers SP van Kann DH Stafleu A Candel MJ Dagnelie PC Thijs C de Vries NK Interaction between physical environment, social environment, and child characteristics in determining physical activity at child care Health Psychol 2011 30 84 90 21133542 10.1037/a0021586

[B28] Bower JK Hales DP Tate DF Rubin DA Benjamin SE Ward DS The childcare environment and children’s physical activity Am J Prev Med 2008 34 23 29 10.1016/j.amepre.2007.09.022 18083447

[B29] Hannon JC Brown BB Increasing preschoolers’ physical activity intensities: an activity-friendly preschool playground intervention Prev Med 2008 46 532 536 10.1016/j.ypmed.2008.01.006 18313741

[B30] Gubbels JS van Kann DH Jansen MW Play equipment, physical activity opportunities, and children’s activity levels at childcare J Environ Public Health 2012 2012 326520 22811736 10.1155/2012/326520 PMC3395256

[B31] Boldemann C Blennow M Dal H Martensson F Raustorp A Yuen K Wester U Impact of preschool environment upon children’s physical activity and sun exposure Prev Med 2006 42 301 308 10.1016/j.ypmed.2005.12.006 16448688

[B32] Brown WH Pfeiffer KA McIver KL Dowda M Addy CL Pate RR Social and environmental factors associated with preschoolers’ nonsedentary physical activity Child Dev 2009 80 45 58 10.1111/j.1467-8624.2008.01245.x 19236392 PMC2648129

[B33] Cardon G van Cauwenberghe E Labarque V Haerens L de Bourdeaudhuij I The contribution of preschool playground factors in explaining children’s physical activity during recess Int J Behav Nutr Phys Act 2008 5 11 10.1186/1479-5868-5-11 18302741 PMC2266778

[B34] Bradley RH Evans GW, Wachs TD From home to day care: Chaos in the family/child-care mesosystem Chaos and its Influence on children’s Development 2010 Washington: American Psychological Association

[B35] Bronfenbrenner U The Ecology of Human Development: Experiments by Nature and Design 1979 Cambridge, MA: Harvard University Press

[B36] Kelly JG Changing contexts and the field of community psychology Am J Community Psychol 1990 18 769 792 10.1007/BF00938064

[B37] Scarr S Developmental theories for the 1990s: Development and individual differences Child Dev 1992 63 1 19 10.2307/1130897 1343618

[B38] Brug J van Lenthe FJ Kremers SP Revisiting Kurt Lewin: how to gain insight into environmental correlates of obesogenic behaviors Am J Prev Med 2006 31 525 529 10.1016/j.amepre.2006.08.016 17169715

[B39] Swinburn B Egger G Raza F Dissecting obesogenic environments: the development and application of a framework for identifying and prioritizing environmental interventions for obesity Prev Med 1999 29 563 570 10.1006/pmed.1999.0585 10600438

[B40] Ding D Gebel K Built environment, physical activity, and obesity: what have we learned from reviewing the literature Health Place 2012 18 100 105 10.1016/j.healthplace.2011.08.021 21983062

[B41] Vereecken C Huybrechts I Meas L De Henauw S Food consumption among preschoolers. Does the school make a difference? Appetite 2008 51 723 726 10.1016/j.appet.2008.04.013 18514366

[B42] Ball SC Benjamin SE Ward DS Dietary intakes in North Carolina child-care centers: are children meeting current recommendations? J Am Diet Assoc 2008 108 718 721 10.1016/j.jada.2008.01.014 18375233

[B43] Bruening KS Gilbride JA Passannante MR McClowry Dietary intake and health outcomes among young children attending 2 urban day-care centers J Am Diet Assoc 1999 99 1529 1535 10.1016/S0002-8223(99)00375-2 10608946

[B44] Vereecken C Huybrechts I van Houte H Martens V Wittebroodt I Maes L Results from a dietary intervention study in preschools “Beastly Healthy at School” Int J Public Health 2009 54 142 149 10.1007/s00038-009-8028-2 19296055

[B45] Williams CL Bollella MC Strobino BA Spark A Nicklas TA Tolosi LB Pittman BP “Healthy-start”: outcome of an intervention to promote a heart healthy diet in preschool children J Am Coll Nutr 2002 21 62 71 10.1080/07315724.2002.10719195 11838889

[B46] Zuercher JL Kranz S Toddlers and preschoolers consume more dietary fiber when high-fiber lunch items are served Child Obes 2012 8 71 75 22799483 10.1089/chi.2011.0054

[B47] Alhassan S Sirard JR Robinson TN The effects of increasing outdoor play time on physical activity in Latino preschool children Int J Pediatr Obes 2007 2 153 158 10.1080/17477160701520108 17852547

[B48] Cardon G Labarque V Smits D de Bourdeaudhuij I Promoting physical activity at the pre-school playground: the effects of providing markings and play equipment Prev Med 2009 48 335 340 10.1016/j.ypmed.2009.02.013 19236894

[B49] Dowda M Pate RR Trost SG Almeida MJ Sirard JR Influences of preschool policies and practices on children’s physical activity J Community Health 2004 29 183 196 15141894 10.1023/b:johe.0000022025.77294.af

[B50] Dowda M Brown WH McIver KL Pfeifer KA O’Neill JR Addy CL Pate RR Policies and characteristics of the preschool environment and physical activity of young children Pediatrics 2009 123 e261 e266 10.1542/peds.2008-2498 19171578 PMC2632768

[B51] Dowda M Pfeiffer KA Brown WH Mitchell JA Byun W Pate RR Parental and environmental correlates of physical activity of children attending preschool Arch Pediatr Adolesc Med 2011 165 939 944 10.1001/archpediatrics.2011.84 21646573

[B52] McKenzie TL Sallis JF Elder JP Berry CC Hoy PL Nader PR Zive MM Broyles SL Physical activity levels and prompts in young children at recess: a two-year study of a bi-ethnic sample Res Q Exerc Sport 1997 68 195 202 10.1080/02701367.1997.10607998 9294873

[B53] Gunter KB Rice KR Ward DS Trost SG Factors associated with physical activity in children attending family child care homes Prev Med 2013 54 131 133 22178820 10.1016/j.ypmed.2011.12.002

[B54] Nicaise V Kahan D Sallis JF Correlates of moderate-to-vigorous physical activity among preschoolers during unstructured outdoor play periods Prev Med 2011 53 309 315 10.1016/j.ypmed.2011.08.018 21878351

[B55] Hustyi KM Normand MP Larson TA Morley AJ The effect of outdoor activity context on physical activity in preschool children J Appl Behav Anal 2012 45 401 405 10.1901/jaba.2012.45-401 22844146 PMC3405934

[B56] van Cauwenberghe E de Bourdeaudhuij I Maes L Cardon G Efficacy and feasibility of lowering playground density to promote physical activity and to discourage sedentary time during recess at preschool: a pilot study Prev Med 2012 55 319 321 10.1016/j.ypmed.2012.07.014 22846504

[B57] Tucker P van Zandvoort MM Burke SM Irwin JD The influence of parents and the home environment on preschoolers’ physical activity behaviours: a qualitative investigation of childcare providers’ perspectives BMC Public Health 2011 11 168 10.1186/1471-2458-11-168 21414218 PMC3070650

[B58] Lloyd-Williams F Bristow K Capewell S Mwatsama M Young children’s food in Liverpool day-care settings: a qualitative study of pre-school nutrition policy and practice Public Health Nutr 2011 14 1858 1866 10.1017/S1368980011000619 21557874

[B59] Fees B Trost S Bopp M Dzewaltowski DA Physical activity programming in family child care homes: providers’ perceptions of practices and barriers J Nutr Educ Behav 2009 41 268 273 10.1016/j.jneb.2008.01.013 19508932

[B60] Wilke S Opdenakker C Kremers SPJ Gubbels JS Factors influencing child care workers’ promotion of physical activity in children aged 0–4 years: a qualitative study Early Years 2013 epub 25 July 2013

[B61] O’Connor JP Temple VA Constraints and facilitators for physical activity in family day care Austr J Early Childh 2005 30 4 1 9

[B62] Copeland KA Sherman SN Kendeigh CA Kalkwarf HJ Saelens BE Societal values and policies may curtail preschool children’s physical activity in child care centers Pediatrics 2012 129 265 274 10.1542/peds.2011-2102 22218842 PMC3269117

[B63] Johnson SL Ramsay S Armstrong Shultz J Branen LJ Fletcher JW Creating potential for common ground and communication between early childhood program staff and parents about young children’s eating J Nutr Educ Behav 2013 45 558 570 10.1016/j.jneb.2013.02.009 23769298

[B64] Koplan JP, Liverman CT, Kraak VI Preventing Childhood Obesity: Health in the Balance 2005 Washington DC: The National Academy Press 22379642

[B65] Baumgartner JJ McBride B Exploring parental philosophies regarding childcare: overlap orientations and the influence of childcare programs on families Early Child Dev Care 2009 179 931 947 10.1080/03004430701635434

[B66] Tavecchio L van Ijzendoorn R Stams GJ Onderzoek: kinderopvang en thuis twee gescheiden werelden Kinderopvang 1996 12 24 27

[B67] Kontos S Peters DL, Kontons S The attitudinal context of family-daycare relationships Continuity and Discontinuity of Experience in Child Care 1987 Norwood, NJ: Ablex 91 113

[B68] van IJzendoorn MH Tavecchio LWC Stams GJ Verhoeven M Reiling E Attunement between parents and professional caregivers: a comparison of childrearing attitudes in different child-care settings J Marriage Family 1998 60 771 781 10.2307/353545

[B69] Feagans LV Manlove EE Parents, infants, and day-care teachers: interrelations and implications for better child-care J Appl Dev Psychol 1994 15 585 602 10.1016/0193-3973(94)90024-8

[B70] Shpancer N The home-daycare link: mapping children’s new world order Early Childh Res Q 2002 17 374 392 10.1016/S0885-2006(02)00170-9

[B71] Perry CK Garside H Morones S Hayman LL Physical activity interventions for adolescents: an ecological perspective J Prim Prev 2012 33 111 135 10.1007/s10935-012-0270-3 22760973

[B72] Kremers SP de Bruijn GJ Droomers M van Lenthe F Brug J Moderators of environmental intervention effects on diet and activity in youth Am J Prev Med 2007 32 163 172 10.1016/j.amepre.2006.10.006 17197152

[B73] de Bruijn GJ Kremers SP de Vries H van Mechelen W Brug J Associations of social-environmental and individual-level factors with adolescent soft drink consumption: results from the SMILE study Health Educ Res 2007 22 227 237 16880219 10.1093/her/cyl066

[B74] Gubbels JS Kremers SP Stafleu A Dagnelie PC Goldbohm RA de Vries NK Thijs C Diet-related restrictive parenting practices. Impact on dietary intake of 2-year-old children and interactions with child characteristics Appetite 2009 52 423 429 10.1016/j.appet.2008.12.002 19114065

[B75] Gubbels JS Kremers SP Stafleu A de Vries SI Goldbohm RA Dagnelie PC de Vries NK van Buuren S Thijs C Association between parenting practices and children’s dietary intake, activity behavior and development of body mass index: the KOALA Birth Cohort Study Int J Behav Nutr Phys Act 2011 8 18 10.1186/1479-5868-8-18 21401954 PMC3065396

[B76] Judd CM McClelland GH Culhane SE Data analysis: continuing issues in the everyday analysis of psychological data Annu Rev Psychol 1995 46 433 465 10.1146/annurev.ps.46.020195.002245 7872734

[B77] Friederichs SA Kremers SP Lechner L de Vries NK Neighborhood walkability and walking behavior: the moderating role of action orientation J Phys Act Health 2013 10 15 22 10.1123/jpah.10.4.515 22975667

[B78] Rech CR Reis RS Hino AAF Hallal PC Personal, social and environmental correlates of physical activity in adults from Curitiba, Brazil Prev Med 2014 58 53 57 24220100 10.1016/j.ypmed.2013.10.023

[B79] Bracy NL Millstein RA Carlson JA Conway TL Sallis JF Saelens BE Kerr J Kelli KL Frank LD King AC Is the relationship between the built environment and physical activity moderated by perceptions of crime and safety? Int J Behav Nutr Phys Act 2014 11 24 10.1186/1479-5868-11-24 24564971 PMC3942774

[B80] Ball SC Benjamin SE Hales DP Marks J McWilliams CP Ward DS The Environment and Policy Assessment and Observation (EPAO) Child Care Nutrition and Physical Activity Instrument 2005 Chapel Hill: Center for Health Promotion and Disease Prevention, University of North Carolina at Chapel Hill

[B81] Brown WH Pfeiffer KA McIver KL Downda M Almeida MJ Pate RR Assessing preschool children’s physical activity: the observational system for recording physical activity in children-preschool version Res Q Exerc Sport 2006 77 167 176 16898273 10.1080/02701367.2006.10599351

